# Clinical, Histological, and Immunohistochemical Findings in Inclusion Body Myositis

**DOI:** 10.1155/2018/5069042

**Published:** 2018-01-29

**Authors:** Leonardo Valente de Camargo, Mary Souza de Carvalho, Samuel Katsuyuki Shinjo, Acary Souza Bulle de Oliveira, Edmar Zanoteli

**Affiliations:** ^1^Departamento de Neurologia, Faculdade de Medicina da Universidade de São Paulo, São Paulo, SP, Brazil; ^2^Disciplina de Reumatologia, Faculdade de Medicina da Universidade de São Paulo, São Paulo, SP, Brazil; ^3^Setor de Doenças Neuromusculares, Disciplina de Neurologia, Universidade Federal de São Paulo (UNIFESP), São Paulo, SP, Brazil

## Abstract

Sporadic inclusion body myositis (sIBM) is considered the most common acquired myopathy aged over 50 years. The disease is characterized by a particular process of muscle degeneration characterized by abnormal deposit of protein aggregates in association with inflammation. The aim of this study was to present clinical and muscle histopathological findings, including immunostaining for LC3B, p62, *α*-synuclein, and TDP-43, in 18 patients with sIBM. The disease predominated in males (61%) and European descendants, with onset of clinical manifestations around 59 years old. The most common symptoms were muscle weakness, falls, dysphagia, and weight loss. Hypertension was the main comorbidity. Most of the cases presented with paresis predominantly proximal in lower limbs and distal in upper limbs. Immunosuppressive treatment showed to be not effective. Muscle histological findings included dystrophic changes, endomysial inflammation, increased lysosomal activity, and presence of rimmed vacuoles and of beta-amyloid accumulation, in addition to high frequency of mitochondrial changes. There was increased expression of LC3B, p62, *α*-synuclein, and TDP-43 in muscle biopsies. The sIBM has characteristic clinical and histological findings, and the use of degeneration and autophagic markers can be useful for the diagnosis.

## 1. Introduction

Sporadic inclusion body myositis (sIBM) is a late-onset form of myopathy classified in the group of inflammatory myopathies. It is considered the most common form of myopathy in patients over 50 years of age, with a prevalence of 3,5 in 100,000 individuals and a male/female ratio of 3 : 1 [1, 2]. Whereas 18–20% of patients develop symptoms before 60 years of age, it is important to consider the diagnosis of sIBM in all patients with consistent symptoms after 30 years old [3, 4]. Clinically, the disease affects predominantly the quadriceps and the gastrocnemius muscles in the lower limbs and the finger flexors in the upper limbs [5–8]. The skeletal muscle abnormalities include an endomysial inflammatory reaction in association with degenerative changes characterized by the presence of rimmed vacuoles, intracytoplasmic inclusions formed by the accumulation of abnormal proteins, *β*-amyloid deposits, and mitochondrial changes [5, 9, 10]. The presence of such degenerative alterations suggests that the disease might be actually a form of muscular degeneration with an associated inflammatory reaction. This would explain the absence of a response to immunosuppressive therapy [11, 12]. However, the precise relationship between degenerative and inflammatory mechanisms is still not clear, and several lines of researches have shown that the inflammatory process could induce or even worsen the degeneration [13].

Some of the degenerative abnormalities include abnormal deposition of protein aggregates formed from amyloid precursor protein (APP), *β*-amyloid 42, phosphorylated tau (tau-p), *α*-synuclein, *α*-B-crystallin, clusterin, presenilin 1, gelsolin, apolipoprotein E (APOE), *γ*-tubulin, and numerous proteins related to oxidative stress, among them the heat shock proteins [10–15]. Interestingly, there are many similarities between the degenerative changes observed in sIBM and in Alzheimer's and Parkinson's diseases. These anomalies include abnormal accumulation of congophilic inclusions and many proteins with similar posttranslational modifications (i.e., *α*-synuclein, p62, TDP-43, LC3B, and ubiquitin), in association with inhibition of 26S proteasome and autophagic systems (defective lysosomal degradation) [11–15]. The inhibition of these two degradative systems contributes to the formation of the protein aggregates (nondegraded), amyloid accumulation, and cytoplasmic vacuolization [11, 12].

Due to its slow progression and unspecific results in blood tests, diagnosis of sIBM is frequently delayed, and in many cases the main initial diagnoses include polymyositis or neurogenic disorders. The most commonly used specific diagnostic criteria for sIBM were published by Griggs et al. in 1995 [16]. However, in several patients with typical clinical sIBM the muscle biopsies do not have the histopathologic findings. In an attempt to increase the sensitivity of diagnosis, several new criteria were proposed: (1) diagnostic criteria of the European Neuromuscular Center (ENMC) to sIBM in 1997 [17]; (2) diagnostic criteria of Neuromuscular Disease Center at the Medical Research Council (MRC) in 2008/2009 to sIBM [18, 19]; and more recently (3) diagnostic criteria of ENMC to sIBM-2011 [20]. In a recent study, Brady et al. [21] found that the criteria proposed by ENMC-2011 were the most sensitive to sIBM, diagnosing 88% of cases, compared with 76% of Griggs criteria and 27% of ENMC-1997 criteria [21]. This same group of researchers suggested a flowchart diagnosis for sIBM based on pathologic findings [22]. From 24 diagnostic categories for sIBM proposed since 1987, Lloyd et al. [23] identified 12 categories with very high specificity of 97% or more, but some had precarious sensitivity as low as 11%. The best performing category was ENMC-2011 (probable) with a sensitivity of 84% [23].

This study aims to present clinical and skeletal muscle histological findings in 18 patients with sIBM. Additionally, autophagy and neurodegeneration markers were applied in the muscle biopsies to detect immunohistochemical abnormalities that could be potentially useful in differentiating sIBM from other forms of inflammatory myopathies.

## 2. Patients and Methods

### 2.1. Patients and Clinical Evaluation

We evaluated 18 patients with clinical and histological diagnosis of sIBM from two of the largest neuromuscular centers in the city of São Paulo, Brazil, during the period of 2013 to 2016. Diagnostic criteria proposed by Griggs et al. [16] were used for the inclusion of patients in the study. Patients with a family history of hereditary IBM and with any other neuromuscular disease were excluded. This study was approved by the Ethics Committee for Research Project of our institution.

Patients were evaluated according to general clinical history, age of onset of symptoms, initial symptoms, evolution of the symptoms, and the presence of depressive symptoms, disturbance of equilibrium, dysphagia, dyspnea, and weight loss. Patients were asked about family history and previous use of medications. The presence of weakness was assessed through neurological examination, including assessment using the Medical Research Council (MRC) scale of muscle strength and motor functional condition using the modified Rankin scale [24]. The effects of functional motor deficits under activities of daily living (ADL) were assessed by the simplified Barthel index [25]. Most patients were evaluated on more than one occasion with an interval of at least 6 months.

Ancillary examinations done in different laboratories or services were recorded, including serum creatine phosphokinase (CK) measurement, rheumatologic tests, tumor markers, virus serology, and electromyography/nerve conduction study (EMG/NCS).

### 2.2. Muscle Biopsy and Staining Reactions

All patients had muscle biopsy examination performed between 2004 and 2016 (Table 4). Biopsies 1a, 4a, 5a, 16, 17, and 18 were performed at the deltoid muscle; 1b, 2, 7b, 8, 9, 11b, and 15 were performed at biceps brachii muscle, and the others were performed at vastus lateralis. The methodology used for processing the biopsies followed standard procedures: after they are removed, muscle fragments are snap frozen in isopentane frozen in liquid nitrogen and stored in a freezer at −80°C before processing. The fragments are sequentially sectioned in coronal position in cryostat at a temperature of −25°C, with a thickness from 6 to 8 microns. The frozen sections were stained with hematoxylin and eosin (HE), modified Gomori trichrome, periodic acid-Schiff (PAS), and “oil red” O (ORO). In addition, the histochemical reactions, NADH-tetrazolium reductase (NADH-TR), succinate dehydrogenase (SDH), cytochrome c oxidase (COX), adenosine triphosphatase (ATPase) in pH acid (4.3 and 4.6) and alkaline (9.3), and acid phosphatase, were done. The reactions were performed according to techniques well established in the literature [26].

The slides were analyzed qualitatively by light microscopy according to the variability in fiber size, increase in endomysial/perimysial connective tissue, proportion of fibers with nuclear centralization, and the presence of fibers with necrosis and/or macrophagy, regenerating fibers, inflammation (endomysial, perimysial, and perivascular), vacuolar formation, rimmed vacuoles, and mitochondrial alterations (RRF: ragged red fibers, SDH-positive fibers, and COX-negative fibers). In addition, the presence of neurogenic alterations (angulated fibers, fiber type grouping), internal cytoarchitecture changes (oxidative defects, minicores, and moth-eaten and lobulated fibers), and lipids and glycogen accumulation were assessed. These aspects were evaluated qualitatively as present (P) or absent (A), as well as quantitatively, according to mild (+), moderate (++), and severe (+++) degrees. Congo-red reaction was also carried out in alkaline pH, useful in identifying amyloid deposits, via immunofluorescence microscopy with a Texas red filter [27].

### 2.3. Immunohistochemical Reactions

The frozen sections were subjected to immunohistochemistry using polymer amplification system Novolink (Novolink kit max polymer detection system, Novocastra®, RE7140 RE7150-K or K-code, Newcastle upon Tyne, UK) followed by staining with DAB (diaminobenzidine). The slides with muscle samples remained for 10 minutes at room temperature to dry, and sections were incubated in a blocking solution at room temperature (Peroxidase Block Novocastra®, RE7101 code, Newcastle upon Tyne, UK) for 5 minutes. After washing in TBS three times for 5 minutes, the slides were incubated with the primary antibodies diluted in the second blocking buffer (Protein Block) for 1 hour at room temperature. The slides were washed two times in TBS of 5 minutes and incubated with primary antibody blocking solution (Post Primary, Novocastra®, RE7111 code, Newcastle upon Tyne, UK) for 20 minutes, followed by washing in TBS two times for 5 minutes and incubation with polymer (polymer Novolink™, Novocastra®, RE7112 code, Newcastle upon Tyne, UK) for 20 minutes. After washing in TBS two times for 5 minutes, the reactions were revealed using DAB (diaminobenzidine) [50 uL DAB Chromogen-diluted RE7105 code 1 mL Novolink™ DAB Substrate Buffer-Polymer-RE7143 code Novocastra®, Newcastle upon Tyne, UK] for about 2 to 5 minutes and subsequently rinsed in running water two times for 2 minutes to block DAB, followed by dripping with Harris hematoxylin for counter-staining and finally dehydration and mount sections. The primary antibodies used were anti-CD68 (Mouse/EBM11, DAKO/M0718, 1 : 100), anti-CD4 (Mouse/4B12, DAKO/M7310, 1 : 100), anti-CD8 (Mouse/C8/144B, DAKO/IS623, 1 : 100), anti-MHC-I (Mouse/W6/32, DAKO/M0736, 1 : 100), anti-LC3B (Rabbit, Sigma/L7543, 1 : 100), anti-*α*-synuclein (Rabbit, Sigma/S3062, 1 : 100), anti-p62/SQSTM1 (Rabbit, Sigma/P0068, 1 : 100), anti-phospho TDP-43 (Mouse/11-9, Cosmo Bio/pS409/410, 1 : 5000), anti-*γ* Sarcoglycan (Mouse/35DAG/21B5, Novocastra/ab49811, 1 : 100), anti-*α* Sarcoglycan (Mouse/Ad1/20A6, Novocastra/NCL-L-a-SARC, 1 : 100), anti-dystrophin 1 (rod domain) (Mouse/Dy4/6D3, Novocastra/NCL-DYS1, 1 : 100), anti-dysferlin (Mouse/Ham3/17B2, Novocastra/NCL-Hamlet-2, 1 : 100), and anti-caveolin-3 (Rabbit, Abcam/ab2912, 1 : 100). To standardize the immunohistochemical reactions, muscle biopsy from a patient with normal histological diagnosis (case 19), two patients with histological diagnosis of dermatomyositis (cases 20 and 21), and two with histological diagnosis of polymyositis (cases 22 and 23) were used. The individual with normal histological diagnosis was submitted to muscle biopsy due to clinical complaint of fatigue.

### 2.4. Statistical Analysis

For the statistical calculation, we used the Pearson and Spearman coefficients, considering a relationship to be statistically significant when *p* < 0.05.

## 3. Results

### 3.1. Clinical Findings

It was observed that sIBM was predominant in males (61% of cases). Approximately 83% (*n* = 15) of patients were European descendants. The average age of onset of symptoms was 58.8 ± 10.9 years old (ranging from 38 to 75 years old). In 17% of patients (*n* = 3), the symptoms began before the age of 45, in two men (40 and 42 years old) and one female (38 years old). In 56% of cases, the symptoms appeared after 60 years of age (Table 1).

The average time between the onset of symptoms and the sIBM diagnosis was 7.4 ± 7.1 years (ranging from 11 months to 31 years). One patient (case 16), after a period of treatment for inflammatory myopathy, was diagnosed with motor neuron disease (MND) and later with diagnosis of sIBM after muscle biopsy.

The most common symptoms were muscle weakness, postural instability with falls, dysphagia, and weight loss. Dysphagia was a common symptom found in 67% of patients (*n* = 12) (Table 1). Weight loss was observed in nine patients (50%). Thirteen patients (72%) had complained of frequent falls, often interpreted by patients as disturbance of equilibrium, and in six of them this complaint was present in the first year of the manifestations (Table 1). Dyspnea was reported by 28% (*n* = 5) of patients (Table 1). One of the patients had obstructive sleep apnea and chronic obstructive pulmonary disease (COPD) secondary to smoking, and he was using home oxygen therapy. Two other patients also had COPD, but with spirometry demonstrating restrictive disorder. Case 4 had resting dyspnea complaints, but normal spirometry, while Case 5 had asthma. The presence of dyspnea had a moderate correlation with worse motor function (*p* < 0.04), greater ADL limitations (*p* < 0.02), and no apparent relation to the time of disease evolution.

The main comorbidities observed in this study were hypertension (*n* = 13/72%), diabetes mellitus type 2 (*n* = 4/22%), osteopenia/osteoporosis (*n* = 4/22%), dyslipidemia (*n* = 4/22%), hyperuricemia/gout (*n* = 4/22%), COPD (*n* = 3/17%), benign prostatic hyperplasia (*n* = 3/17%), and glaucoma without family history (*n* = 3/17%). Other conditions observed in our patients were hypertriglyceridemia, colonic diverticulosis, cataract, acute myocardial infarction, obstructive sleep apnea syndrome, hypothyroidism, erythematosus tumidus (lymphocytic infiltration of Jessner), herpes zoster, radical prostatectomy, Wolf-Parkinson-White, restless legs syndrome, conservative treatment for herniated lumbar disc, surgical treatment for lumbar disc herniation, ocular toxoplasmosis, ischemic stroke, prior HTLV-I infection, squamous cell carcinoma (face), sarcoidosis, migraine with aura, coronary insufficiency, asthma, total hysterectomy (myoma), peripheral neuropathy (diabetic), pulmonary hypertension, idiopathic cirrhosis, interstitial pneumopathy, pulmonary thromboembolism, central retinal vein thrombosis, and nephrolithiasis.

**Figure 1:**
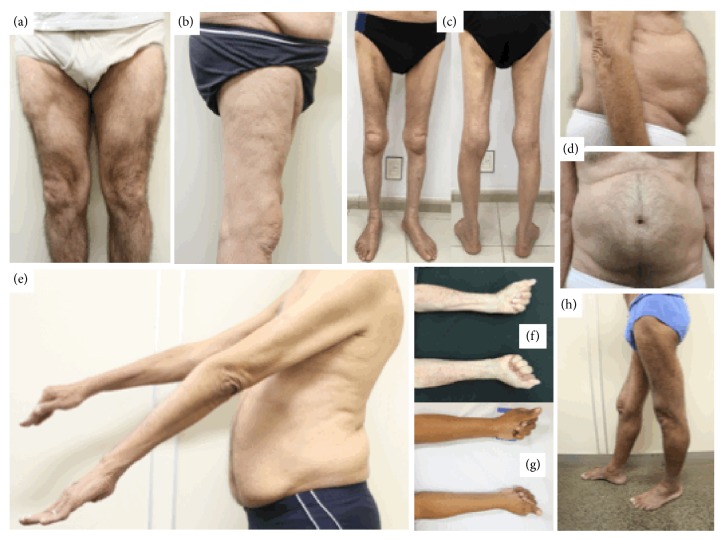
Clinical findings of patients with sIBM. Intense atrophy of quadriceps femoris muscle in case 4 (a) and case 1 (b), global atrophy of lower limbs in case 16 (c), Beevor's signal in case 2 (d), atrophy of upper limbs in case 2 (e), atrophy of finger flexors in cases 16 (f) and case 7 (g), and bilateral atrophy of quadriceps femoris in case 7 (h).

The muscular involvement pattern was characterized by paresis predominantly proximal in lower limbs and distal in upper limbs (Figure 1). The measurement of muscle strength of patients according to the MRC scale is shown in Table 2. All sIBM patients had involvement of the wrists flexors (except case 13), hand fingers flexors, and leg extensors muscles. Other muscle groups involved in most cases were the biceps brachii, triceps brachii, thigh flexors (particularly the iliopsoas muscle), thigh extensors, and leg flexor muscles. The flexors of the feet were affected in 56% of cases. One interesting finding observed in the case 2 was the weakness of the lower abdominal muscles, accompanied by Beevor's sign described as upper deviation of the umbilicus in the evaluation of abdominal skin reflex (Figure 1(d)).

The functional status of patients is displayed in Table 1, in which the results of two assessments with an interval of 12 to 18 months are presented. We only found statistical correlation between the time of disease progression and the Barthel Index (*p* = 0.033), demonstrating the highest degree of dependence in ADL with the evolution of sIBM. All other relationships were not statistically significant. Some cases were evaluated only one time (cases 4, 13, 15, 16, 17, and 18). In all other cases, worsening of disability and increase of limitations of ADL were observed after 12 to 18 months of the first evaluation. Immunosuppression was not effective over the long term in most of the patients with sIBM. Fifteen patients (83%) had the initial diagnosis of inflammatory myopathy, undergoing several medical treatments described in Table 3.

### 3.2. Ancillary Exams

The absolute values of the highest levels of CK were among 214 U/L and 2,656 U/L, and the relative values for the upper limit of normality (ULN) ranged from 1,11 to 9,48 × ULN.

EMG/NCS was performed in all cases. The patterns found in 12 patients (67%) were polyphasic potentials to slight contraction, paradoxical interference potential to maximal contraction, rest-intense activity characterized by fibrillation, and the presence of motor unit potential with increased amplitude and long duration. Reduction in sensory and/or motor nerve conduction velocities, excluding the compressive mononeuropathies (e.g., carpal tunnel syndrome, ulnar nerve at the elbow, and the common peroneal nerve at the fibular head) were found in 33% of cases. Case 16 held two EMG/NCS, and the first was interpreted as motor neuron disease. Surface electromyography was performed in two patients with dysphagia, one had normal findings (case 5) and the other (case 4) had normal findings at rest, but with increased muscle contraction in laryngeal extrinsic muscles of swallowing saliva. This finding is related to the transition of the pharyngeal phase to the esophageal phase, showing a mild to moderate oropharyngeal dysphagia. Another patient, who also had dysphagia, underwent nasolaryngofibroscopy that showed esophageal-laryngeal reflux (case 2).

### 3.3. Histological and Histochemical Analysis

Of the 18 cases, seven were submitted to two procedures (1a, 1b, 4a, 4b, 5a, 5b, 7a, 7b, 10a, 10b, 11a, 11b, 12a, and 12b), with a total of 25 examined biopsies. The information about the biopsied muscle, biopsy date, and the initial histological diagnosis is presented in Table 4.

**Figure 2:**
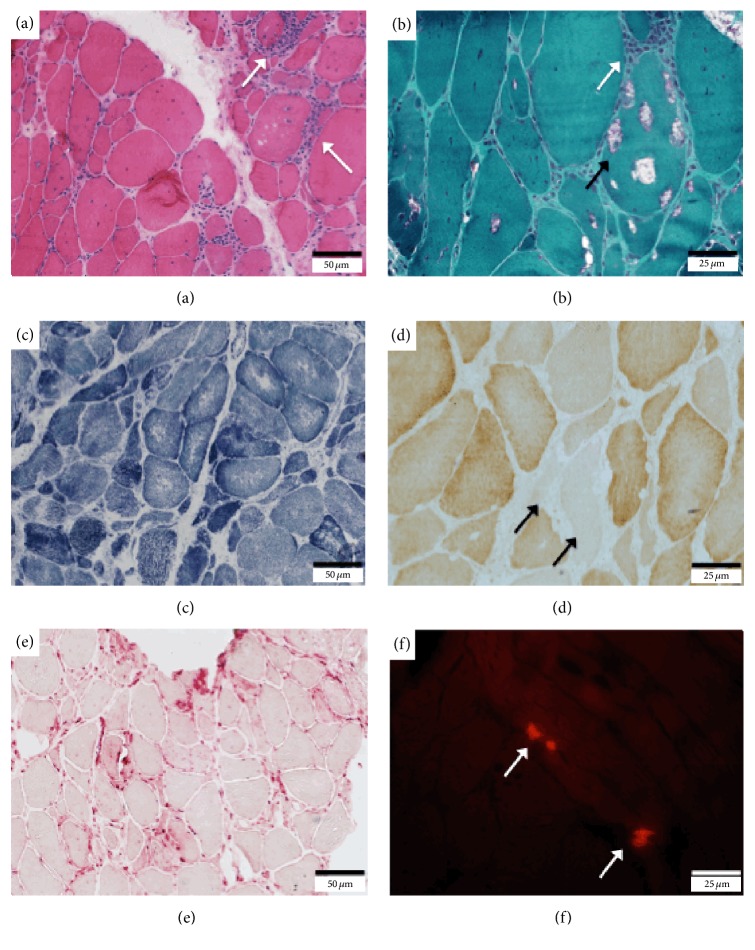
Histological findings in muscle biopsy of patients with sIBM. (a) Endomysial inflammatory reaction (arrows) and global dystrophic pattern (case 4(b)) (H&E). (b) Endomysial inflammatory reaction (white arrow) and rimmed vacuoles (black arrow) (case 4(b)) (Gomori's trichrome). (c) Oxidative defects (case 7(b)) (NADH). (d) Cytochrome C oxidase deficiency (arrows) (case 7(b)). (e) Intense acid phosphatase reaction (case 6). (f) Positive Congo-red staining (arrows) (case 6).

**Figure 3:**
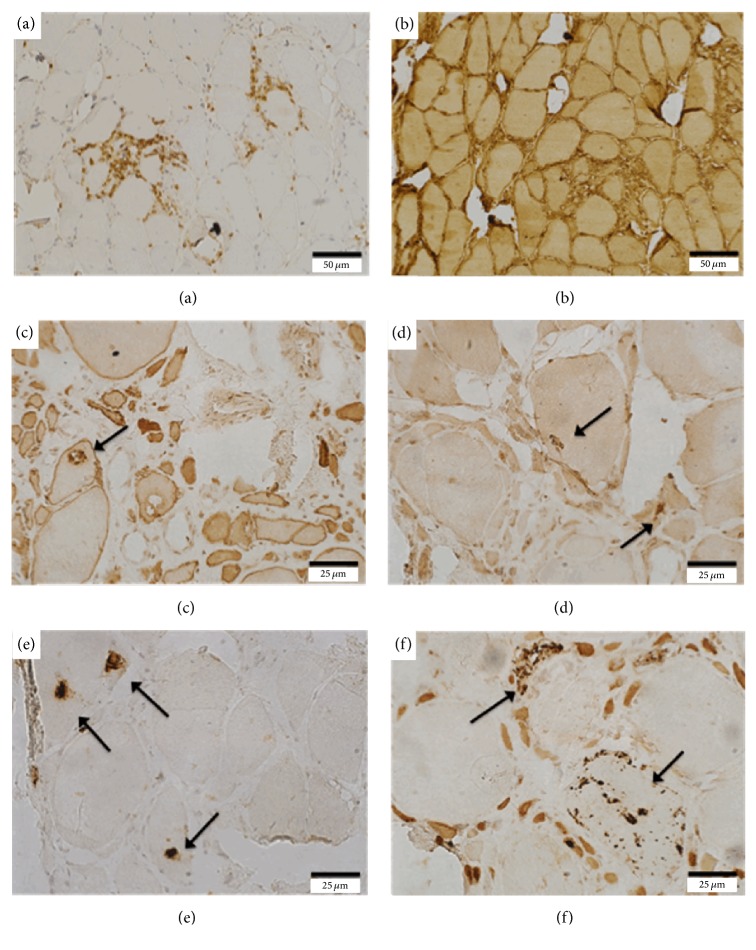
Immunohistochemical findings in muscle biopsy of patients with sIBM. (a) Presence of areas of positive staining for CD8-lymphocytes (case 2). (b) Diffuse increase of histocompatibility antigen type I expression (case 2). (c) Increased LC3 expression in the vacuole (arrow) (case 5(b)). (d) Increased alpha-synuclein expression in the vacuoles (arrows) (case 5(b)). (e) Increased TDP-43 expression in the vacuoles (arrows) (case 5(b)). (f) Increased p62 expression in the vacuoles (arrows) (case 5(b)).

Representative images of histological and immunohistochemical findings of the sIBM cases are shown in Figures 2 and 3. All sIBM biopsies showed preservation of the fiber-typing distribution pattern, variation in size of the fibers, presence of rimmed vacuoles (except biopsies 1a, 4a, 7a, and 12a), increased nuclear internalization, endomysial inflammatory infiltration, increased reaction to acid phosphatase, and unspecific changes at oxidative stains. Just over half of the biopsies showed reduced labeling for COX or COX-negative fibers. Four biopsies had RRF, and ten showed SDH-positive fibers. Most of biopsies showed signs of necrosis with increased macrophagy and increased endomysial and perimysial connective tissue (Table 4). All cases had no signs of lipid or glycogen accumulation. All cases of sIBM showed signs of beta-amyloid accumulation with positive Congo-red reaction (Figure 2(f)/Table 4). Congo-red reaction was not performed in four biopsies (7a, 10a, 11a, and 12a) due to lack of stored material.

### 3.4. Immunohistochemical Analysis

All sIBM and inflammatory myopathy control cases showed increased labeling for inflammatory markers (CD4, CD8, CD68, and MHC-I) (Figures 3(a) and 3(b)/Table 5). There was increased expression of LC3B (autophagic marker), especially in the vacuoles, in 67% of sIBM cases (*n* = 12) (Figure 3(c)). The expression was not increased in cases 6, 9, 12, 14, 17, and 18, as well as in the controls (cases 19, 20, 21, 22, and 23). The expression of p62/SQSTM1 (anti-p62) was positive in all cases of sIBM (Figure 3(f)). Expression of *α*-synuclein staining was positive in about 89% of sIBM cases (Figure 3(d)). The expression of anti-phospho TDP-43 (anti-TDP-43) was positive in all cases with sIBM (Figure 3(e)), with no reaction in the controls. The positivity for anti-TDP-43 was predominantly in vacuoles, while positivity for anti-*α*-synuclein occurred both in the vacuoles and in the nucleus of some muscle fibers. Immunohistochemical reaction to membrane proteins (dystrophin and sarcoglycans), dysferlin, and caveolin-3 was normal in all tested cases. Immunohistochemical reactions for LC3B, TDP-43, p62, and *α*-synuclein were not performed in four biopsies (7a, 10a, 11a, and 12a), as well as inflammatory markers in three biopsies (10a, 11a, and 12a) due to lack of stored material.

### 3.5. Comparison between the Main sIBM Diagnostic Criteria

All sIBM cases included in this study met the criteria proposed by Griggs et al. [16], as well as the diagnostic criteria of the* ENMC-1997* for sIBM [17] and Neuromuscular Diseases Center of the Medical Research Council (MRC) 2008/2009 [19]. But when the diagnostic criteria proposed by the ENMC in 2011 [20] were used, three cases (cases 1, 9, and 10) could not be included because the patients started the symptoms before they were 45 years old.

## 4. Discussion

We present here clinical and skeletal muscle pathological findings of patients with sIBM who met the classic diagnostic criteria proposed by Griggs et al. [16]. In this study, sIBM predominated in males (61% of cases), similar to the other studies in the literature [1, 2, 28], with onset of clinical manifestations at 58.8 years of age (±10.9). Forty-four percent of patients had symptoms before the sixth decade of life; other studies have indicated that about 18–20% of patients with sIBM develop symptoms before that age [29]. Furthermore, three patients (17%) had symptoms before 45 years, which excludes these patients from the inclusion criteria of the ENMC-2011 for sIBM, even considering that these patients have presented all clinical and histological findings from the other criteria frequently used by others (Griggs-1995, ENMC-1997, and MRC-2008/2009). Recently described as the diagnostic criteria with higher sensitivity and specificity [21, 23], the ENMC-2011 excludes patients with early age of 45 years even with clinical and pathological confirmation. Thus, a change in ENMC-2011 criteria—that is, considering age > 35 years old and not >45 years old as in the original publication—would increase the diagnostic sensitivity for sIBM in our study from 83% to 100%. These facts would occur in several other series, including a recent Chinese report that included a patient with an onset of symptoms at 38 years old and in which 11% of patients were between 40 and 49 years old [28]. However, it should be considered that an early onset of the manifestations implies a more detailed investigation into other conditions that may mimic sIBM, such as muscular dystrophies, myofibrillar myopathies, and inflammatory myopathies of other etiologies. A critical analysis of the sIBM criteria was recently published and the authors stated that any currently accepted diagnostic criteria will be shown to have “missed” patients with atypical features [30].

The identification of cN1A antibodies in sIBM might be useful to increase the diagnosis specificity [31–33]. Despite some studies have indicated that moderate reactivity was 70% sensitive and 92% specific and high reactivity was 34% sensitive and 98% specific for the diagnosis of sIBM [31–33], such autoantibodies are also found in autoimmune rheumatic diseases (dermatomyositis, systemic lupus erythematosus, systemic sclerosis, Sjögren's syndrome, and polymyositis) [34]. Unfortunately, in our country, this antibody is not yet widely available, so it was not possible to include this data in this study. Certainly, future sIBM criteria would consider the inclusion of more specific newly recognized autoantibodies to increase the specificity of the diagnosis.

In our study, the average time between the onset of symptoms and the sIBM diagnosis was 7.4 ± 7.1 years. These findings point out that in our country, as noted in many other series, sIBM was underdiagnosed. In addition, 83% of our patients had the initial diagnosis of polymyositis. These cases were submitted to severe drug treatments already demonstrated to be ineffective for sIBM, often causing considerable side effects. One of the factors that most confuses the diagnosis is the EMG/NCS interpretation. Some sIBM cases presented EMG/NCS reports suggesting polymyositis. One of our cases was diagnosed by EMG/NCS as polymyositis and was treated with immunosuppression. In this patient, the EMG/NCS was repeated and the diagnosis was changed to motor neuron disease. Many patients get the diagnosis of motor neuron diseases because they present, together with the electrophysiological misinterpretation, asymmetric distal motor predominance in the upper limbs, dysphagia, dyspnea, weight loss, and dysphonia in some cases [35]. EMG/NCS is an examiner-dependent method and can often hinder the diagnosis and generate unnecessary treatments. Eventually, studies with quantitative EMG/NCS could improve the sensitivity of electrophysiological diagnostic of sIBM.

In our study, the most frequent complaints of the patients regarding early symptoms were weakness of one or more extremities, presented in 94% of our cases. Most commonly, patients reported bilateral involvement of the lower limbs (39%), followed by noncharacterized weakness in the four limbs (28%) and initial weakness in one of the lower limbs (17%). One patient presented onset of symptoms with dysphagia, another with simultaneous weakness in the upper limbs, and another with initial weakness in one of the upper limbs. This predominance for weakness in the proximal muscles of the lower limbs is well demonstrated in the literature [4, 23].

Another frequent complaint of patients (72%) in our study was related to postural instability during gait and falls from height, which was often interpreted as disturbance of equilibrium; just under half of the patients cited this as an early symptom, although it has not been described in the literature. Other relevant symptoms were dysphagia (67%), weight loss (50%), and depressive symptoms (33%), the latter preceding or following the onset of the classical symptoms of sIBM, a fact that points to another similarity with neurodegenerative diseases such as Alzheimer and Parkinson diseases and may even arise before other complaints. When the relationship between the Barthel Index and the sIBM development time was analyzed, it was noted that the time to disease progression was inversely proportional to the Barthel index (*p* = 0.033). All other correlations showed no statistically significant relationship, most probably due to the small number of patients. The presence of depressive symptoms and weight loss predominated in the first year after the onset of symptoms.

The major comorbidity associated with sIBM was arterial hypertension (72%), possibly being aggravated in some cases by the side effects of previously prescription drugs, mainly oral corticosteroids. Approximately 22% of patients had diabetes mellitus type 2, osteopenia/osteoporosis, hyperlipidemia, and hyperuricemia/gout, and some of these also were exacerbated by drug therapy. The presence of a large number of comorbidities in these cases indicates the need for extra care when prescribing immunosuppressive drugs. In addition, physicians should reassess the maintenance of immunosuppression when no therapeutic response is noticed.

Interestingly, despite the relationship described between sIBM and serum positivity for antibodies to hepatitis C virus [36–38], none of the patients in this series had positive serology for hepatitis C. In another Brazilian study with 30 patients with sIBM, one patient had positive serology for hepatitis C and two for HIV [29]. Freitas et al. [39] reported a coexistence of sIBM with HIV infection, and Cupler et al. [40] reported two cases with this association. In our study, only one patient had viral seropositive for HTLV-I, which was also reported in an American patient [40]. Another patient developed shingles during the course of the disease and the immunosuppressive treatment.

Although sIBM may present as a paraneoplastic condition, or as coexisting with it, only one patient in our study had prior squamous cell carcinoma in the face. In another Brazilian study with 30 patients with sIBM, four patients had concurrent cancer; two with prostate carcinoma, one with follicular adenocarcinoma ovarian, and the other with breast adenocarcinoma [29].

One case in our study had an interesting history of pulmonary sarcoidosis in 1979, which was treated at the time and remained asymptomatic until then. This patient developed early symptoms of sIBM after about 30 years of the pulmonary event, but with no granulomatous findings on muscle biopsy. Similar cases have been rarely described in the literature [41–45]. Another patient of our series presented histopathologic diagnosis of lupus erythematosus tumidus, lymphocytic infiltration of Jessner in skin biopsy, and no evidence of systemic lupus erythematosus, a situation not described in the literature until now.

We characterized the pattern of muscle impairment in patients with sIBM as paresis with proximal predominance of lower limb (quadriceps femoris and flexor muscles of the thigh) and distal upper limb (flexors of the wrists and hand fingers), a fact well characterized in the literature [4–6]. There was also a significant impairment of the biceps brachii, triceps, iliopsoas, thigh extensors, and leg flexor muscles. Just over half of the patients had involvement of the foot flexor muscles, most commonly seen in the later stages of the disease, while 17% of patients had weakness in one of the lower limbs as the initial symptom. Such muscle impairment corresponds to the typical pattern described in sIBM [4–6] and is included in the clinical diagnostic criteria.

Only one patient had weakness of the abdominal muscles, predominantly in the bilateral lower level, with the presence of upward deviation of the umbilicus in the evaluation of skin-abdominal reflex bilaterally, also called Beevor's sign. This sign was recently described in sIBM [46].

Our study showed that 67% of patients had dysphagia. A study of Li et al. [28] including 28 patients with sIBM found dysphagia in only 7% of patients. This complaint should be actively questioned by the patient because most of them do not notice the difficulty in swallowing, which may explain the low incidence in other series. In the study of Alverne et al. [29], only 13% of patients had weight loss without apparent cause, while about 40% of cases in our study progressed with weight loss.

A retrospective analysis of immunosuppressive therapies used in some of our cases did not appear to significantly influence motor decline or worsen general symptoms, while four patients with sIBM in this series showed mild, but not sustained improvement. One patient had mild motor improvement, but for less than six months after ten doses of abatacept. Two patients had symptomatic improvement after intravenous methylprednisolone; one with no sustained motor improvement of less than 6 months and the other with partial improvement of dysphagia lasting less than 12 months. Another patient received intravenous methylprednisolone for two days, followed by intravenous immunoglobulin (IVIG) for two days and also showed a slight improvement with motor function worsening after six months. Another patient showed a large drop in CK serum levels during use of prednisone and later with the combination of methotrexate and azathioprine, but without any clinical improvement. In addition to the case described with partial and not sustained motor improvement, two patients received IVIG without any clinically change. A recent publication evaluated six patients undergoing treatment with subcutaneous human immunoglobulin, and all showed improvement in muscle strength and resolution of dysphagia. Four patients got worse before 12 months, and the other two got worse after 12 months [47].

All patients with sIBM showed increased CK serum levels that were 10 times lower than ULN levels (below the limits proposed in the diagnostic criteria) [16, 20, 21].

In our series, seven cases were submitted to repeated muscle biopsy procedures, three of which were done in our service (cases 7, 10, and 12), including cases 7 and 12 for lack of diagnosis and case 10 for an unknown reason (as the patient already had sIBM diagnostic in the first biopsy). One case was assessed with external neurologist and underwent a new procedure for an unknown reason (case 11). In three other patients (cases 1, 4 and 5), two underwent a new muscle biopsy due to a lack of histological diagnosis and one did so for an unknown reason. The interval between biopsies from the same patient ranged from 2 months to 12 years. Three cases were subjected to biopsy in the deltoid muscle, two of which had no strength deficits in this muscle. The best place for a muscle biopsy, if not guided by imaging, is the muscle with impairment of strength combined with no marked atrophy.

All muscle biopsies of the patients presented with normal distribution of fiber types, variable dystrophic changes, endomysial and perimysial inflammatory reaction, rimmed vacuoles, and increased staining for acid phosphatase. Other relevant histological findings were the presence of mitochondrial changes (83%) including the presence of reduced staining for COX, SDH-positive fibers, and RRF. In addition, all biopsies had Congo-red positive staining indicating beta-amyloid accumulation. All the above histological findings are characteristic of sIBM and are indicated at the inclusion criteria.

All cases with sIBM and inflammatory controls were positive for inflammatory markers, which does not help to differentiate between them. In particular, MHC-I and MHC-II are useful markers to characterize inflammatory myopathies in general. In this study we used only MHC-I, but not MHC-II, and an overall increase in the staining was detected in all cases, including in the controls with other forms of inflammatory myopathy. But when the degeneration and autophagy activation markers were used, they were positive in all cases with sIBM, and there was no staining in patients with other types of inflammatory myopathies. Anti-p62 (autophagy) and anti-TDP-43 (degeneration) expressed predominantly in the vacuoles and were positive in all sIBM biopsies. As showed by Askanas and Engel [11], immunostaining with p62 is linked increasingly to early processes of sIBM's pathogenesis, demonstrating the involvement of the accumulation of p-tau in the early stages of the diseases, making our finding of the presence of labeling for p62 in all cases with sIBM a strong indication that this is a good marker.

The development of techniques that allow an early and accurate diagnosis of sIBM with evaluation of serum markers and muscle imaging studies will be important to reduce the need for more invasive tests, such as muscle biopsy.

For now, the gold standard for diagnosis remains the muscle biopsy, combined with the typical clinical pattern. Our study evaluated patients that filled all the clinical criteria described so far. Future studies with patients in the early stages of the disease, or nonspecific symptoms, using the same markers, could demonstrate their sensitivity in early diagnosis of the disease.

## Figures and Tables

**Table 1:** Patient data, modified Rankin Scale (mRS), Barthel Index (BI) and impairment in activities of daily living (ADL) in 18 cases with sIBM.

Case	Sex	Ethnicity	Onset^1^	Evolution^2^	Falls^3^	Dysphagia	WL^4^	Dyspnea	DS	IS	mRS		BI		ADL limitations	
											2013/2014	2015	2013/2014	2015	2013/2014	2015
1	M	E	40	401	**P**	**P**	**P**	**P**	**P**	PLLW	4	4	45	40	moderate	moderate
2	M	E	54	245	**P**	**P**	**A**	**P**	**P**	4LW	3	4	85	55	mild	moderate
3	M	E	52	91	**A**	**P**	**A**	**P**	**P**	PLLW	3	4	75	55	mild	moderate
4	M	E	65	77	**P**	**P**	**P**	**A**	**P**	Dysphagia	2	U	95	U	mild	U
5	M	E	55	89	**P**	**P**	**P**	**A**	**A**	PLLW	2	2	95	85	mild	mild
6	F	E	61	63	**A**	**A**	**A**	**A**	**A**	PLLW	3	3	75	65	mild	mild
7	M	Af	55	91	**P**	**P**	**P**	**A**	**A**	4LW	2	2	100	85	independent	mild
8	F	E	53	125	**P**	**P**	**P**	**A**	**A**	PLLW	4	4	75	55	mild	moderate
9	M	E	42	101	**A**	**A**	**A**	**A**	**A**	DULW	2	2	90	80	mild	mild
10	F	Af	38	99	**A**	**A**	**A**	**P**	**A**	1PULW	3	3	70	60	mild	mild
11	M	E	66	139	**P**	**P**	**P**	**A**	**P**	1DLLW	2	2	95	90	mild	mild
12	M	E	60	39	**P**	**A**	**A**	**A**	**A**	1DLLW	2	2	95	90	mild	mild
13	F	As	55	126	**A**	**P**	**P**	**A**	**A**	4LW	U	2	U	95	U	mild
14	F	E	65	96	**P**	**A**	**P**	**A**	**P**	PLLW	2	2	90	85	mild	mild
15	F	E	75	46	**P**	**P**	**A**	**A**	**A**	4LW	U	2	U	95	U	mild
16	M	E	73	24	**P**	**P**	**P**	**P**	**A**	1DLLW	U	3	U	80	U	mild
17	F	E	64	57	**P**	**P**	**A**	**A**	**A**	4LW	U	2	U	85	U	mild
18	M	E	75	60	**P**	**A**	**A**	**A**	**A**	PLLW	U	4	U	40	U	moderate

^1^Age of onset of symptoms in years. ^2^Time to disease progression in months. ^3^Postural instability or falls. ^4^WL = weight loss preceding or at onset of the disease. F = female. M = male. As = Asian. E = European. Af = African. DS = depressive symptoms. IS: initial symptom. P = present. A = absent. U = undone. PLLW = proximal lower limbs weakness. 4LW = uncharacterized weakness of the 4 limbs. DULW = distal upper limbs weakness. 1PULW = proximal weakness of one lower limb. 1DLLW: distal weakness of one lower limb.

**Table 2:** Evaluation of muscle strength (MRC: Medical Research Council, 1981) in 18 patients with sIBM.

Case	NF	NE	RD	LD	RB	LB	RT	LT	RWF	LWF	RWE	LWE	RHFF	LHFF	RHFE	LHFE	ABD	RTF	LTF	RTE	LTE	RLF	LLF	RLE	LLE	RFF	LFF	RFE	LFE	RFFF	LFFF	RFFE	LFFE
1	4	4	4	4	4	4	4	4	3	3	3	3	3	3	5	5	5	3	3	3	3	3	3	3	3	5	5	5	5	5	5	5	5
2	5	5	5	5	4	4	5	5	3	3	5	5	3	3	5	5	3	3	3	5	5	5	5	4	4	4	4	5	5	5	5	5	5
3	5	5	4	4	4	4	4	4	3	3	4	4	3	3	4	4	5	4	4	4	4	4	4	4	4	3	3	4	4	5	5	5	5
4	5	5	5	5	5	5	5	5	4	4	5	5	4	4	5	5	5	5	4	5	5	5	5	4	4	5	5	5	5	5	5	5	5
5	4	4	5	5	4	4	3	3	4	4	5	5	3	3	5	5	5	3	3	3	3	3	3	3	3	5	4	5	4	5	4	5	4
6	5	5	5	5	4	4	5	5	4	4	5	5	4	4	5	5	5	5	5	5	5	4	4	4	4	5	5	5	5	5	5	5	5
7	5	5	5	5	5	5	5	5	4	4	5	5	4	4	5	5	5	4	4	4	4	4	3	4	3	5	5	5	5	5	5	5	5
8	3	3	4	4	4	4	4	4	3	3	4	4	3	3	4	4	4	2	2	2	2	3	3	2	2	4	4	4	4	4	4	4	4
9	5	5	5	5	4	4	4	4	4	4	5	5	4	4	5	5	5	4	4	5	5	5	5	4	4	5	5	5	5	5	5	5	5
10	5	5	4	4	2	3	3	3	4	4	3	3	3	3	3	3	4	2	2	2	2	4	4	3	3	4	4	4	4	4	4	4	4
11	5	5	4	4	5	4	4	4	4	4	5	5	5	4	4	4	5	5	5	5	5	4	4	4	4	4	4	5	5	4	4	5	5
12	4	4	4	4	4	4	4	4	3	3	5	5	3	3	5	5	5	4	4	4	4	5	5	3	3	4	0	5	5	4	0	5	4
13	4	5	4	4	4	4	4	4	5	5	5	5	4	4	4	4	4	3	3	4	4	4	4	4	4	5	5	4	4	5	5	5	5
14	4	4	4	4	4	4	5	5	4	4	5	5	4	4	5	5	5	4	4	3	4	4	4	3	4	5	5	5	5	5	5	5	5
15	4	4	4	5	4	4	5	5	4	4	5	5	4	4	5	5	5	4	3	4	3	5	5	4	4	5	4	5	5	5	5	5	5
16	4	4	5	5	4	4	3	3	3	2	4	4	3	2	4	4	4	4	4	4	4	3	3	3	2	3	2	4	4	4	4	4	4
17	5	5	5	5	4	4	4	4	4	4	5	5	4	4	5	5	5	4	4	5	5	5	5	4	4	5	5	5	5	5	5	5	5
18	5	5	4	4	4	4	4	4	4	3	5	4	4	3	5	4	4	4	4	4	4	4	4	2	2	3	3	4	4	4	4	4	4

NF = neck flexors. NE = neck extensors. RD = right deltoid. LD = left deltoid. RB = right biceps. LB = left biceps. RT = right triceps. LT = left triceps. RWF = right wrist flexors. LWF = left wrist flexors. RWE = right wrist extensors. LWE = left wrist extensors. RHFF = right hand fingers flexors. LHFF = left hand fingers flexors. RHFE = right hand fingers extensors. LHFE = left hand fingers extensors. ABD = abdominal muscles. RTF = right thigh flexors. LTF = left thigh flexors. RTE = right thigh extensors. LTE = left thigh extensors. RLF = right leg flexors. LLF = left leg flexors. RLE = right leg extensors. LLE = left leg extensors. RFF = right foot flexors. LFF = left foot flexors. RFE = right foot extensors. LFE = left foot extensors. RFFF = right foot fingers flexors. LFFF = left foot fingers flexors. RFFE = right foot fingers extensors. LFFE = left foot fingers extensors. Muscle strength degrees: 5 = normal. 4 = against gravity/light resistance. 3 = only against gravity. 2 = joint movement without overcoming the gravity. 1 = muscle contraction without joint movement. 0 = no muscle contraction.

**Table 3:** Drugs used by patients with sIBM, duration of treatment, side effects, and clinical and laboratory changes related to the treatment.

Case	Drug	Duration	Side effects	Clinical and laboratory changes
1	AZAT 150 mg/day	6 months	Chills + prostration	No
	PRED 60 mg/day	8 years	No	No
	PRED 60 mg 8/8 hours	90 days	Anasarca	No
	Mofetil	3 months	Diarrhea + consumptive syndrome	No
	MTX 20 mg/week	3 years	No	No

2	PRED 60 mg/day	9 days	Anasarca	No

3	IVIG 400 mg/kg	PT 2 days (for 2x)	No	No
	Abatacept	10 doses	No	Light motor improvement NS
	Leflunomide 20 mg/day	3 years	No	No
	PRED 30 mg/day	6 years	No	No
	Cyclophosphamide	1 year	No	No
	AZAT until 250 mg/day	1 year	Leukopenia	No
	Cyclosporine	2x pulse therapy	No	No

4	Deflazacort 45 mg/day	1 month	Weakness worsens	No
	Deflazacort 30 mg/day	4 months	No	No

5	MTX 7,5 mg/week	4 years	No	No

6	PRED 60 mg/day	1 year	No	No
	AZAT 250 mg/day + PRED 20 mg/day	2 months	No	No
	MP 1 gram	PT for 2 days	No	Light motor improvement NS

8	AZAT		Pancytopenia	No
	Cyclosporine		Incoercible vomit	No
	PRED 40 mg/day	3 years	Drug Cushing's syndrome	No
	MP 1 grama	PT for 2 days	No	Partial improvement of dysphagia
	MTX	1 year	Gastric intolerance	No

10	AZAT 150 mg/day	1.5 years	No	No
	MTX 20 mg/week + PRED 60 mg/day	2 years	No	No
	Rituximab	1 cycle	No	No

11	MP 1 gram	PT 5 days	No	No
	PRED 40 mg/day	6 months	No	Reduction CK/no improvement
	MTX + AZAT	4 years	No	Reduction CK/no improvement

12	IVIG 400 mg/kg	2 years	No	No

13	PRED (maximum dose 80 mg/day)	8 years	Edema + weight gain	No
	MP 1 grama + IVIG 400 mg/kg	PT for 2 days/each	No	Light motor improvement NS
	AZAT	1.5 years	No	No

14	MTX	1 month	Gastric intolerance	No

16	MTX	1 year	No	No
	Coenzyme-Q10	15 months	No	No
	Riluzole	15 months	No	No

17	PRED 20 mg/day	6 months	No	No

18	PRED 60 mg/day + folic acid 5 mg/day	9 months	Edema + weight gain	Light motor improvement NS

AZAT = azathioprine. PRED = prednisone. MTX = methotrexate. IVIG = intravenous human immunoglobulin. MP = methylprednisolone. CK = creatine phosphokinase. NS = nonsustained. PT = pulse therapy.

**Table 4:** General data, histological, immunohistochemical and deposits of amyloid (Congo-red) of muscle biopsies in 18 cases with sIBM and controls.

Case	Biopsy date^1^	Histologic diagnoses	Fiber size variation	Necrosis	Macrophagy	RV	AAF	CC	Connective tissue increase		NC	Inflammatory infiltrates			IAPR	Mitochondrial changes			Congo-red
									EMy	PMy		EMy	PMy	PV		COX−	SDH+	RRF	
1																			
a	02/11	UM	+	A	P	A	P	P	+	+	+	P	A	A	+	P	P	A	P
b	02/13	IBM	++	P	A	P	P	P	++	++	+	P	A	A	+	A	P	A	P
2	09/12	IBM	++	P	A	P	A	P	++	++	++	P	P	A	++	A	A	A	P
3	04/12	IBM	++	P	P	P	P	P	+	+	++	P	P	A	++	P	P	P	P
4																			
a	09/11	UM	+	P	A	A	A	P	+	+	++	P	P	A	++	P	P	A	P
b	11/13	IBM	++	P	A	P	A	P	+	+	++	P	A	A	+	A	A	A	P
5																			
a	12/10	IBM	+	P	P	P	A	P	++	+	+	P	P	A	+	P	P	A	P
b	06/14	IBM	+++	A	A	P	A	P	+++	+++	+	P	A	A	++	P	P	A	P
6	02/14	IBM	++	A	A	P	A	P	+	+	++	P	A	A	++	A	A	A	P
7																			
a	10/13	DIST	+++	A	A	A	A	P	+++	+++	++	P	A	A	+++	A	A	A	U
b	03/14	IBM	+++	P	P	P	P	P	++	++	++	P	A	A	++	P	A	A	P
8	02/14	IBM	++	A	A	P	A	P	+	+	++	P	A	A	++	A	A	A	P
9	04/10	IBM	++	P	A	P	P	P	+	A	+	P	P	P	+	A	A	A	P
10																			
a	07/10	IBM	+	A	A	P	A	P	+	+	+	P	P	P	+	A	A	A	U
b	09/13	IBM	+	P	A	P	A	P	+	+	++	P	P	A	++	P	A	A	P
11																			
a	05/04	IBM	+	P	P	P	A	P	+	+	+	P	P	P	+	A	A	A	U
b	01/16	IBM	+	P	P	P	A	P	A	+	+	P	A	A	+	P	P	P	P
12																			
a	05/12	UM	+	P	A	A	P	A	A	A	+	P	A	A	+	A	A	A	U
b	07/12	IBM	+	P	P	P	P	P	A	+	++	P	P	A	+	P	A	A	P
13	10/14	IBM	++	P	P	P	P	P	++	++	++	P	A	A	+	P	A	A	P
14	05/14	IBM	+	P	P	P	P	P	+	A	++	P	A	A	++	A	A	A	P
15	02/15	IBM	+	P	P	P	P	P	+	++	+	P	P	A	+	P	A	A	P
16	10/14	IBM	+	P	P	P	A	P	+	++	+	P	A	A	+	P	P	P	P
17	10/14	IBM	+	A	A	P	P	P	+	++	++	P	A	A	+	A	P	A	P
18	11/14	IBM	+	P	P	P	P	P	+	+	+	P	A	A	++	P	P	P	P
*Controls*																			
19	05/14	NL	A	A	A	A	A	A	+	+	A	A	A	A	A	A	A	A	A
20	03/14	DM	+	A	A	A	P	P	+	+	+	P	P	P	++	A	A	A	A
21	02/14	DM	++	A	A	A	P	P	++	++	+	P	P	P	+	A	A	A	A
22	08/13	PM	+	P	A	A	A	P	A	A	+	P	P	A	++	A	A	A	A
23	08/13	PM	+	P	A	A	A	P	+	+	+	P	P	A	+++	A	A	A	A

IBM = inclusion body myositis. UM = unspecific myopathic changes. DIST = dystrophic pattern. A = absent. P = present. U = unrealized. EMy = endomysial. PMy = perimysial. PV = perivascular. IAPR = increased acid phosphatase reaction. AAF = angulated atrophic fibers. CC = cytoarchitecture changes. NC = nuclear centralization. RV = rimmed vacuoles. ^1^Biopsy date (month/year). +: mild. ++: moderate. +++: severe.

**Table 5:** Immunohistochemical findings of muscle biopsies in 18 cases with sIBM and controls.

Case	CD68	CD4	CD8	MHC-I	LC3B	syn	TDP-43	p62
1								
a	++	+	+	+++	P	P	P	P
b	++	+	+	+	A	P	P	P
2	+++	+	++	+++	P	P	P	P
3	+++	+	+	++	P	P	P	P
4								
a	++	+	+	+++	P	P	P	P
b	+++	+	++	+++	P	P	P	P
5								
a	++	+	+	++	A	P	P	P
b	++	++	++	++	P	P	P	P
6	+++	++	+	++	A	P	P	P
7								
a	+	+	+	++	U	U	U	U
b	++	+	++	++	P	P	P	P
8	+++	+	+	+++	P	P	P	P
9	++	+	+	++	A	P	P	P
10								
a	U	U	U	U	U	P	U	P
b	++	+	++	++	P	P	P	P
11								
a	U	U	U	U	U	U	U	U
b	+	+	+	++	P	P	P	P
12								
a	U	U	U	U	U	U	U	U
b	++	+	+	+++	A	P	P	P
13	++	+	+	++	P	A	P	P
14	++	+	+	++	A	P	P	P
15	++	+	+++	+++	P	P	P	P
16	++	+	+	+++	P	P	P	P
17	++	+	+	+++	A	A	P	P
18	++	++	++	+++	A	P	P	P
*Controls*								
19	++	A	A	A	A	A	A	A
20	A	+	+	++	A	A	A	A
21	++	+	+	+	A	A	A	A
22	+	+	+	+++	A	A	A	A
23	++	+	++	+++	A	A	A	A

A = absent. P = present. U = undone. LC3B = microtubule-associated protein light chain 3. Syn = alpha-synuclein. TDP-43 = transactive response DNA binding protein 43 kDa. P62/SQSTM1 protein = p62/sequestosome 1. MHC-I = main complex histocompatibility type 1. CD4 and CD8 = lymphocytes markers. CD68 = macrophage marker. +: mild. ++: moderate. +++: severe.
